# Predicting the potential distribution areas of *Leptotrombidium rubellum* under current and future climate change

**DOI:** 10.3389/fpubh.2025.1638468

**Published:** 2025-08-07

**Authors:** Qunzheng Mu, Fengfeng Li, Wenyu Li, Xiaoxia Wang, Mingyuan Tang, Kehan Chen, Yihao Jiang, Jingqi Liu, Shirong Zhang, Qiyong Liu, Chuan Wang

**Affiliations:** ^1^West China School of Public Health and West China Fourth Hospital, Sichuan University, Chengdu, Sichuan, China; ^2^State Key Laboratory of Infectious Diseases Prevention and Control, National Institute for Communicable Disease Control and Prevention, Chinese Center for Disease Control and Prevention, Beijing, China; ^3^School of Public Health, Shandong Second Medical University, Weifang, Shandong, China; ^4^Center for Disease Control and Prevention of Ningxia Hui Autonomous Region, Yinchuan, China

**Keywords:** *Leptotrombidium rubellum*, chigger mite, MaxEnt, climate change, potential distribution areas

## Abstract

**Background:**

*Leptotrombidium rubellum (L. rubellum),* a confirmed vector of scrub typhus, was historically restricted to southeastern coastal China but has recently been detected in southwestern regions. Species distribution modeling was applied to predict its current and future potential distribution areas under multiple climate scenarios, identify high-priority surveillance areas, and determine key environmental drivers. The results may facilitate a transition from passive to proactive vector monitoring.

**Methods:**

Fifty-seven potential influencing factors were evaluated. The maximum entropy (MaxEnt) model projected potential distribution areas for near current and future climate scenarios. Occurrence records were extracted from published literature. The selection of environmental variables was conducted using a multi-stage analytical approach, consisting of contribution rate assessment, jackknife tests, and correlation analyses. Model parameters were optimized via feature class and regularization multiplier adjustments.

**Results:**

The MaxEnt model demonstrated high predictive accuracy (AUC = 0.997) with minimal training omission error. July precipitation (prec7) and elevation (elev) were identified as the primary environmental determinants. Projections indicate near current suitable areas are concentrated in southern China, with potential northward expansion under future climate scenarios.

**Conclusion:**

*L. rubellum* exhibits broad distribution areas across China, with climate change likely driving suitable areas expansion. Enhanced surveillance in currently suitable and future at-risk regions is critical to mitigate invasion risks.

## Introduction

Chigger mites serve as the primary vectors of scrub typhus, a febrile illness caused by the obligate intracellular bacterium *Orientia tsutsugamushi*. Emerging epidemiological evidence suggests these mites may serve as competent vectors not only for scrub typhus but also for hemorrhagic fever with renal syndrome (HFRS), epidemic hemorrhagic fever (EHF), and potentially other zoonoses (1–3). Globally, scrub typhus incidence has risen markedly in recent decades, with China representing s one of the most heavily burdened regions (4–6). Notably, *Leptotrombidium rubellum (L. rubellum)* has been epidemiologically confirmed a major scrub typhus vector in multiple Chinese provinces (7, 8), underscoring its public health significance. Traditional entomological surveys initially classified *L. rubellum* as having a narrow distribution, limited to coastal Fujian Province (9). Recent eco-epidemiological investigations, however, have expanded this understanding, confirming *L. rubellum* presence in Yunnan Province and documenting infections across multiple rodent host (9, 10). The distribution patterns of chigger mites are shaped by dynamic interactions between microclimatic conditions, land cover type, and anthropogenic landscape modifications (4, 11, 12). Previous studies indicate that the distribution of *L. rubellum* is influenced by multiple environmental and ecological factors, including temperature, humidity, elevation, land cover type, and host availability (2, 10). Notably, field investigations have revealed that *L. rubellum* exhibits low host specificity and a broad host range, suggesting adaptability across diverse ecological niches. However, the precise mechanisms by which other factors shape its distribution remain insufficiently characterized, warranting further investigation.

Climate change has triggered profound ecosystems transformations over the past century, driving landscape modification, biodiversity loss, and public health impacts through ecological cascades (13–15). Key climatic variables-including temperature regimes, precipitation patterns, and land cover dynamics-directly govern vector distribution, population densities, and pathogen transmission efficiency (16–18). These environmental shifts may f lower ecological barriers for invasive species, potentially enabling range expansion of medically important vectors like *L. rubellum* (2, 19–21). With projected warming trends and precipitation anomalies expected to create increasingly favorable conditions for this scrub typhus vector, predictive modeling of its future geographic range becomes vital for disease surveillance and prevention.

Species distribution models (SDMs) are essential for forecasting species geographic ranges under environmental change. The Maximum Entropy (MaxEnt) algorithm has emerged as a powerful SDM approach, distinguished by its ability to minimize commission errors while offering mechanistic insights. Key advantages include its quantitative assessment of environmental variable contributions, rigorous evaluation of variable importance through jackknife tests, and robust predictive accuracy validation using receiver operating characteristic (ROC) curve analysis (AUC) (22, 23). The MaxEnt model has been widely applied in studies of invasive species, biodiversity conservation, and disease risk assessment (24–28).

This study employed the MaxEnt model to predict current and future potential distribution areas of *L. rubellum* in China based on historical occurrence records, high-resolution bioclimatic variables, elevation data, and land cover type. The relative contributions of environmental factors to observed and projected range shifts were systematically evaluated under four representative climate change scenarios, providing insights for vector control strategies.

## Materials and methods

### Analytical workflow

**Figure 1:**
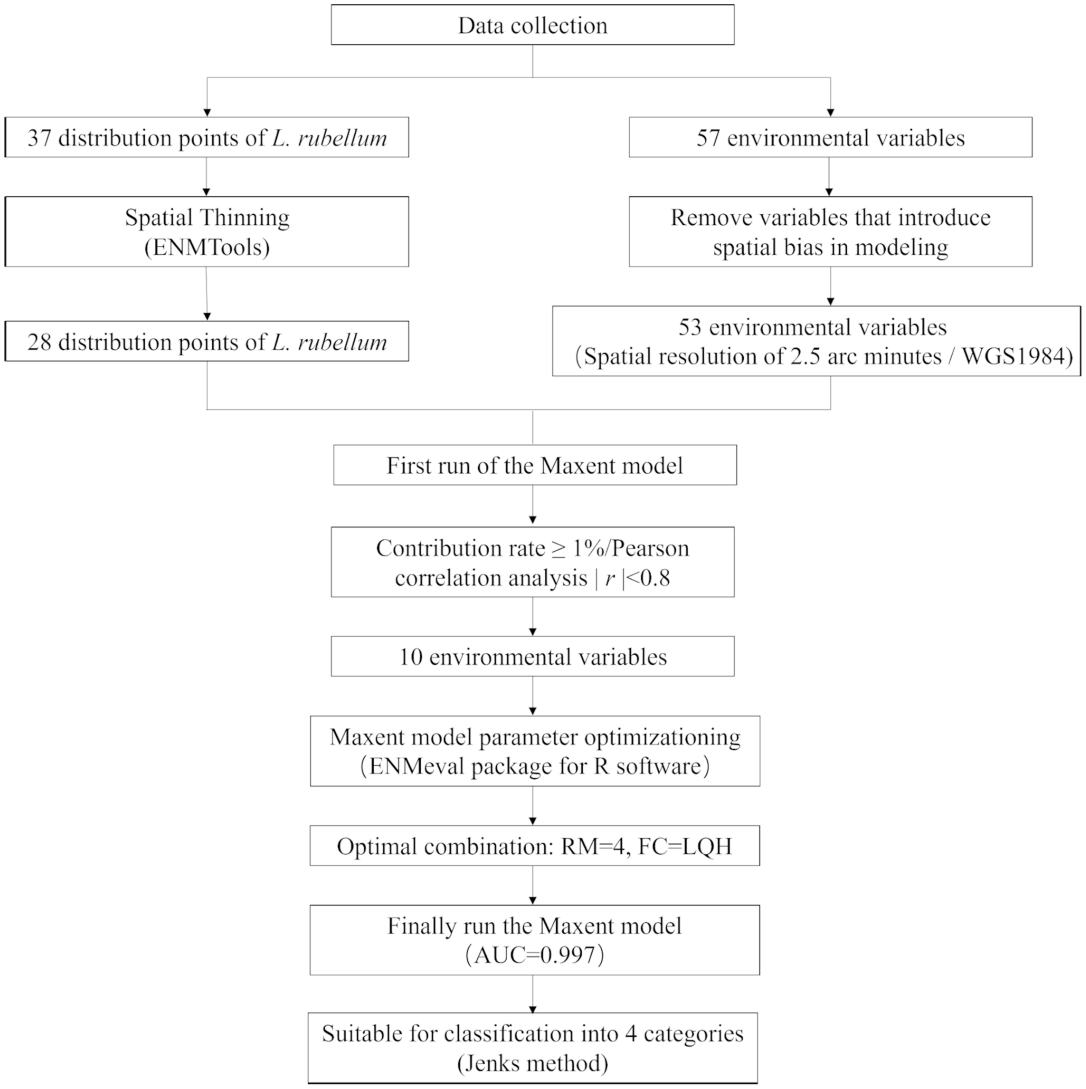
Workflow for predicting potential distribution of *L. rubellum* using MaxEnt model.

This study mainly includes the following parts: collection and screening of *L. rubellum* and related environmental variables, model optimization, and classification of suitable areas (Figure 1).

### Occurrence data collection and processing

**Figure 2:**
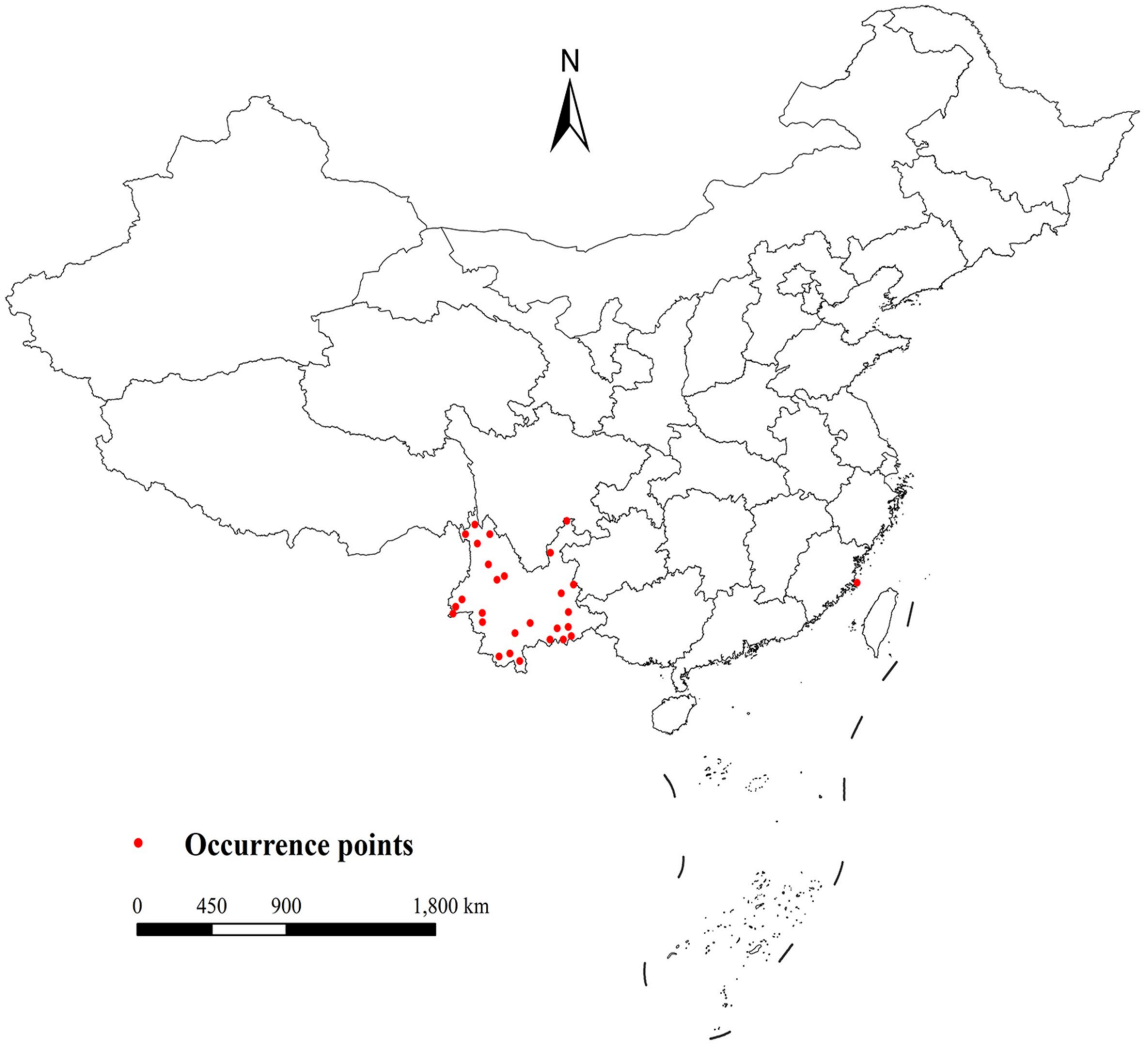
Current distribution of *L. rubellum* in China.

*Leptotrombidium rubellum* occurrence data (1950–2024) were aggregated from Chinese (CNKI, Wan Fang, VIP), international (PubMed, Web of Science, Embase) databases, and related zoological works. We implemented a multi-stage validation process including methodological verification, ecological plausibility assessment, and geographic coordinate standardization. Initial compilation yielded 37 occurrence points (Figure 2; Additional file 1). To address spatial autocorrelation (20), spatial thinning was performed using ENMTools (https://github.com/danlwarren/ENMTools; accessed 20 April 2024), retaining one record per 2.5 arc-minute environmental raster cell. This process resulted in 28 spatially independent occurrence points after removing 9 duplicate records located within identical grid cells.

### Application software and geographic data

The MaxEnt software (version 3.4.1, https://biodiversityinformatics.amnh.org/open_source/maxent/, accessed on 1 March 2024) was employed for species niche and distribution modeling. ArcGIS (version 10.8) was licensed by the Vector Control Department of the Institute of Infectious Disease Control and Prevention, Chinese Center for Disease Control and Prevention. Additional analyses utilized R (version 4.1.0, http://www.r-project.org/, accessed on 1 March 2024) and DIVA-GIS (version 7.5.0, https://diva-gis.org/download.html, accessed on 31 May 2021) for model parameter adjustments. The China map (scale: 1:4,000,000) is from the National Geomatics Center of China (https://www.ngcc.cn/; accessed on 1 April 2024), and the world map (scale: 1:10,000,000) is from the Natural Earth (https://www.naturalearthdata.com/downloads; accessed on 1 April 2024).

### Environmental data processing

Fifty-seven environmental variables were initially compiled, including 56 variables from WorldClim^1^ at 2.5 arc-minute resolution: bioclimatic parameters (bio1–bio19), monthly temperature extremes (tmax1–tmax12; tmin1–tmin12), monthly precipitation (prec1–prec12), and elevation (elev). The dataset encompassed both current (1970–2000) and future climate scenarios (2021–2,100) generated by BCC-CSM2-MR under four SSPs (1–2.6, 2–4.5, 3–7.0, 5–8.5). Land cover projections (2020–2100) at 1 km spatial resolution were obtained from Figshare (https://figshare.com/articles/dataset/Global_LULC_projection_dataset_from_2020_to_2100_at_a_1km_resolution/23542860 LULC projection dataset from 2020 to 2,100 at a 1 km resolution/ 23,542,860). Each GeoTIFF file contains integer raster attribute values from 1 to 6, representing different land use types: cropland, forest, grassland, urban areas, barren lands, and water bodies. All variables were resampled to 2.5 arc-minutes in WGS1984 using ArcGIS 10.8.

### Model optimization

**Figure 3:**
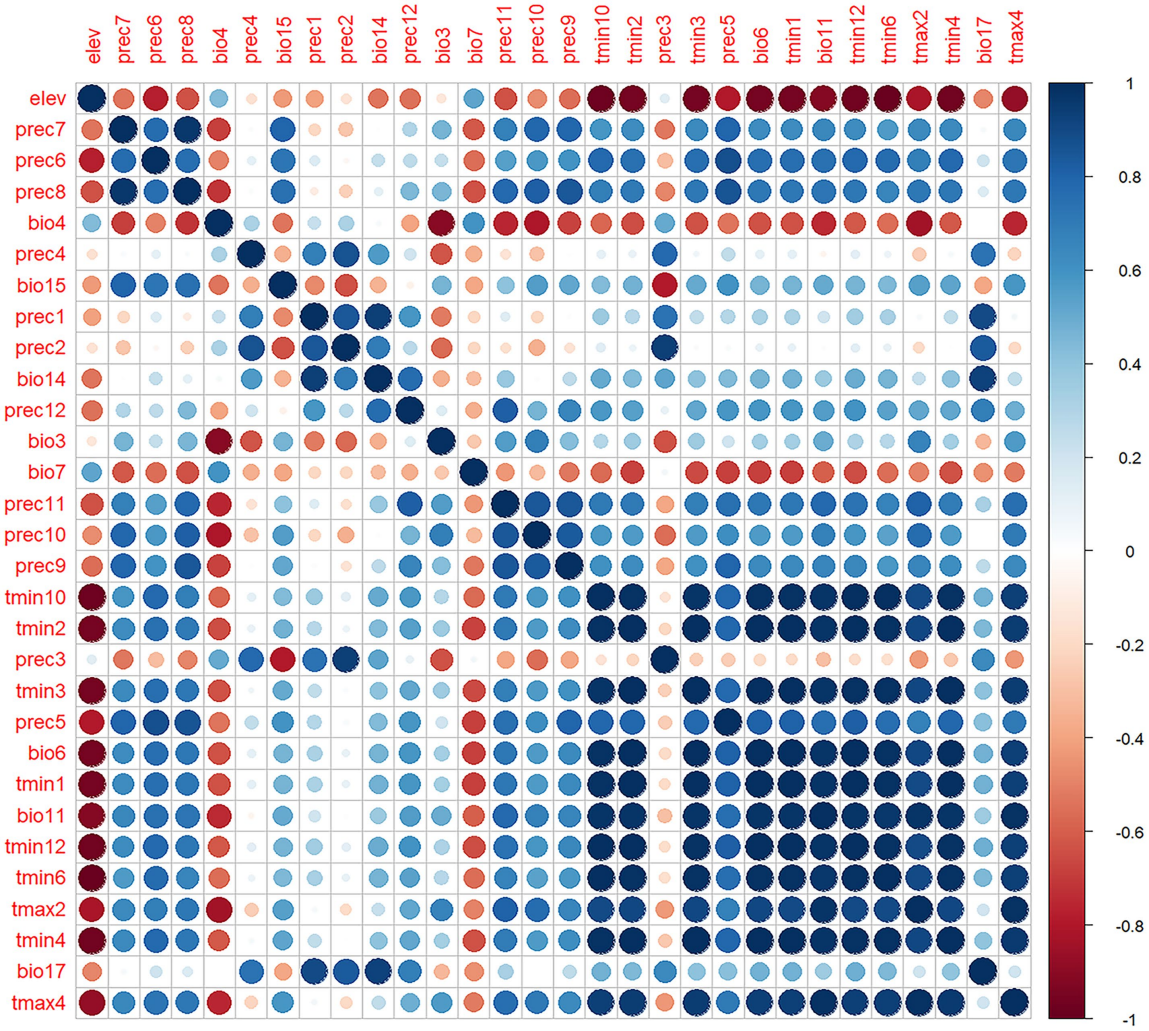
Pearson correlation analysis of environmental variables. The blue dots in the figure represent positive correlation, the red dots represent negative correlation, and the darker the color, the stronger the correlation.

Four bioclimatic variables (bio8, bio9, bio18, bio19) were excluded due to known spatial bias risks (23), yielding 53 variables for initial analysis. MaxEnt 3.4.1 was configured with Cloglog output format, 5,000 bootstrap repetitions, 20 replicates, 75% training and 25% testing data partitioning, and Minimum training presence threshold rule. Variable selection involved first eliminating variables with <1% relative importance, followed by Pearson correlation analysis (|r| ≥ 0.8) to identify redundant variables while retaining those with higher contribution rates (Figure 3), ultimately resulting in 10 optimal climate factors for final modeling (Table 1).

**Table 1:** Environment variables in projecting the distribution of *L. rubellum*.

Variables	Description	Unit	Contribution (%)
prec7	Precipitation in July	mm	26.1
elev	Elevation	m	25.8
prec6	Precipitation in June	m	14.4
bio4	Temperature seasonality (standard deviation × 100)	\	9.1
prec4	Precipitation in April	mm	8.8
prec1	Precipitation in January	mm	5.0
bio15	Precipitation Seasonality	\	3.7
bio7	Temperature Annual Range	°C	3.5
prec12	Precipitation in December	mm	2.6
lc	Land cover	\	1.0

When species distribution points are limited, default model parameters may yield suboptimal performance (29, 30). The initial Jackknife analysis utilized default MaxEnt parameters to identify broadly important variables, while the final model incorporated ENMeval-optimized feature combination (FC) and regularization multiplier (RM) combinations to maximize predictive performance (20). Parameter optimization was conducted using R software, systematically evaluating RM across eight levels (0.5–4 in 0.5-unit increments) and five fundamental FC combinations: linear (L), quadratic (Q), hinge (H), product (P), and threshold (T), generating eight distinct feature sets (L, LQ, LQP, QHP, LQH, LQHP, QHPT and LQHPT). For MaxEnt model parameter optimization, this study used current climate data (1970–2000) from WorldClim as the baseline. Future climate projections (2021–2,100), derived from the BCC-CSM2-MR model under four SSP scenarios (SSP1-2.6, SSP2-4.5, SSP3-7.0, and SSP5-8.5), were applied exclusively for subsequent distribution modeling and were not involved in model parameter calibration.

**Figure 4:**
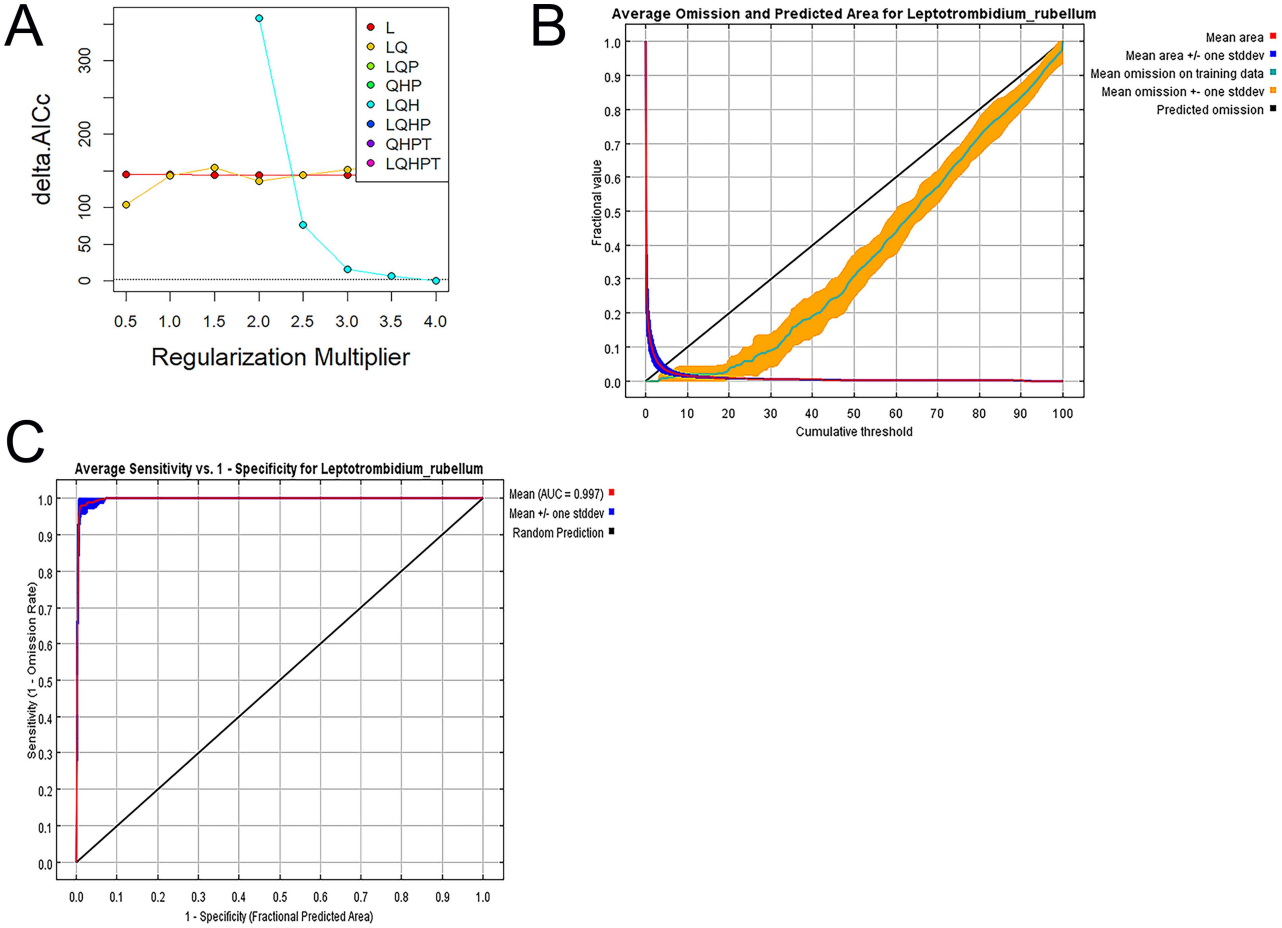
**(A)** AICc values for different regularization multiplier and feature combination. Delta. **(B)** Average omission and predicted area for *L. rubellum*. **(C)** Receiver operating characteristic curve of model prediction results.

Model selection was performed using the ENMeval package in R 4.1.0, with Akaike’s Information Criterion (AIC) as the primary evaluation metric. The optimal model was identified as the one with the lowest AICc value. We calculated delta AICc (ΔAICc) as the difference between each candidate model’s AICc and the minimum AICc value among all models: ΔAICcᵢ = AICcᵢ − min (AICc). Models with ΔAICc < 2 were considered to have substantial empirical support. Alternative models were systematically evaluated through these AICc comparisons (27). This process determined LQH as the optimal FC and 4 as the ideal RM setting (Figure 4A).

Model performance was evaluated through receiver operating characteristic (ROC) curve analysis, with the area under the curve (AUC) serving as the accuracy metric: 0.5–0.7 (low predictive ability), 0.7–0.9 (moderate), and 0.9–1.0 (high). Under current climate conditions, the test set’s average omission rate matched the training set’s (Figure 4B), with training and test dataset AUC values of 0.997 and 0.991, respectively, (Figure 4C). The minimal metric differences and ENMeval-driven parameter optimization confirmed the model’s robustness against overfitting (20, 27).

### Classification of the suitable areas

The distribution probability results of suitable areas generated by the MaxEnt model under current environmental conditions were classified into four categories using the Jenks natural breaks method (21): unsuitable area (0–0.0706), low suitable area (0.0706–0.2745), moderate suitable area (0.2745–0.6313), and high suitable area (0.6313–1). To ensure consistency in evaluation across time periods, these same classification thresholds were systematically applied to categorize habitat suitability under all future climate scenarios.

## Results

### The relationship between the distribution of *Leptotrombidium rubellum* and the environmental variables

**Figure 5:**
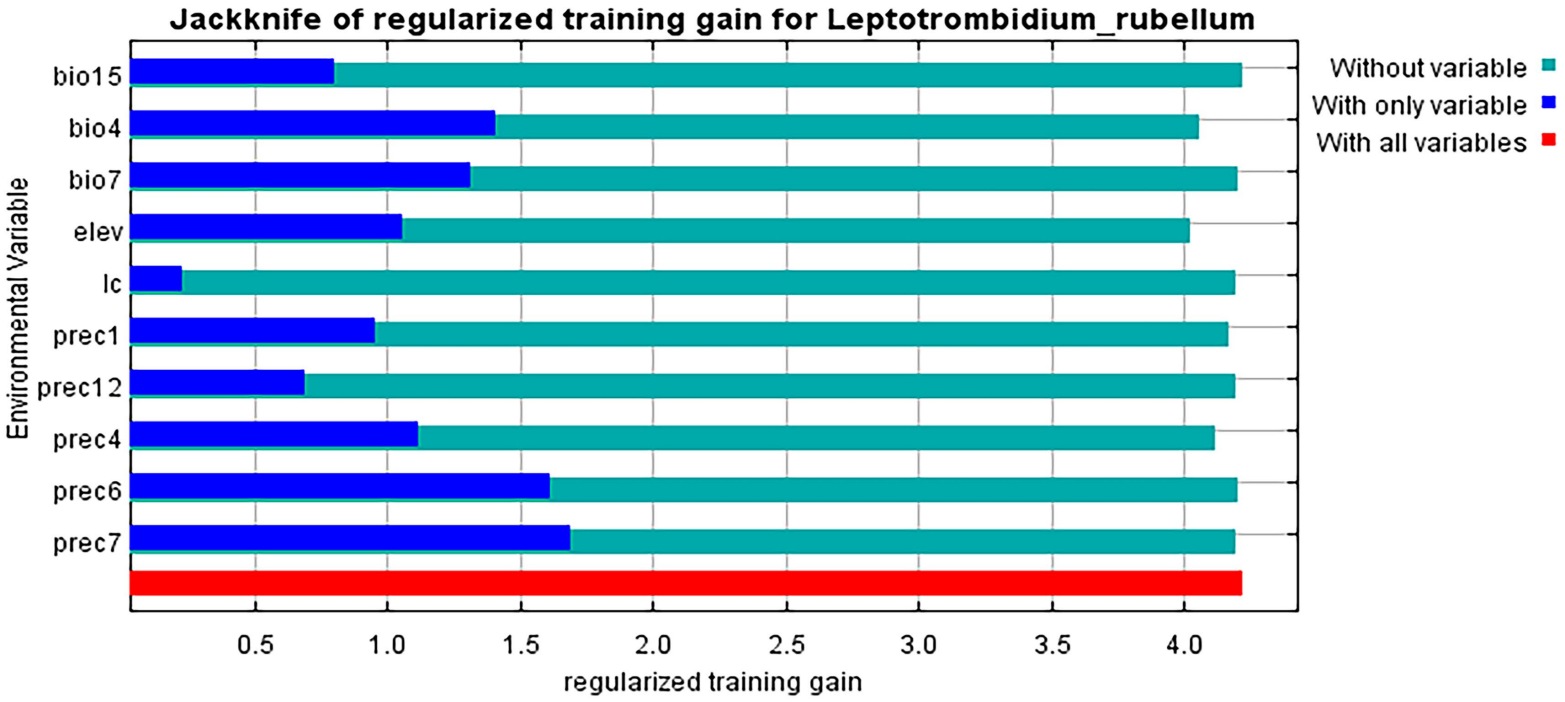
Importance of the influence of environmental variables on the distribution of *L. rubellum*.

**Figure 6:**
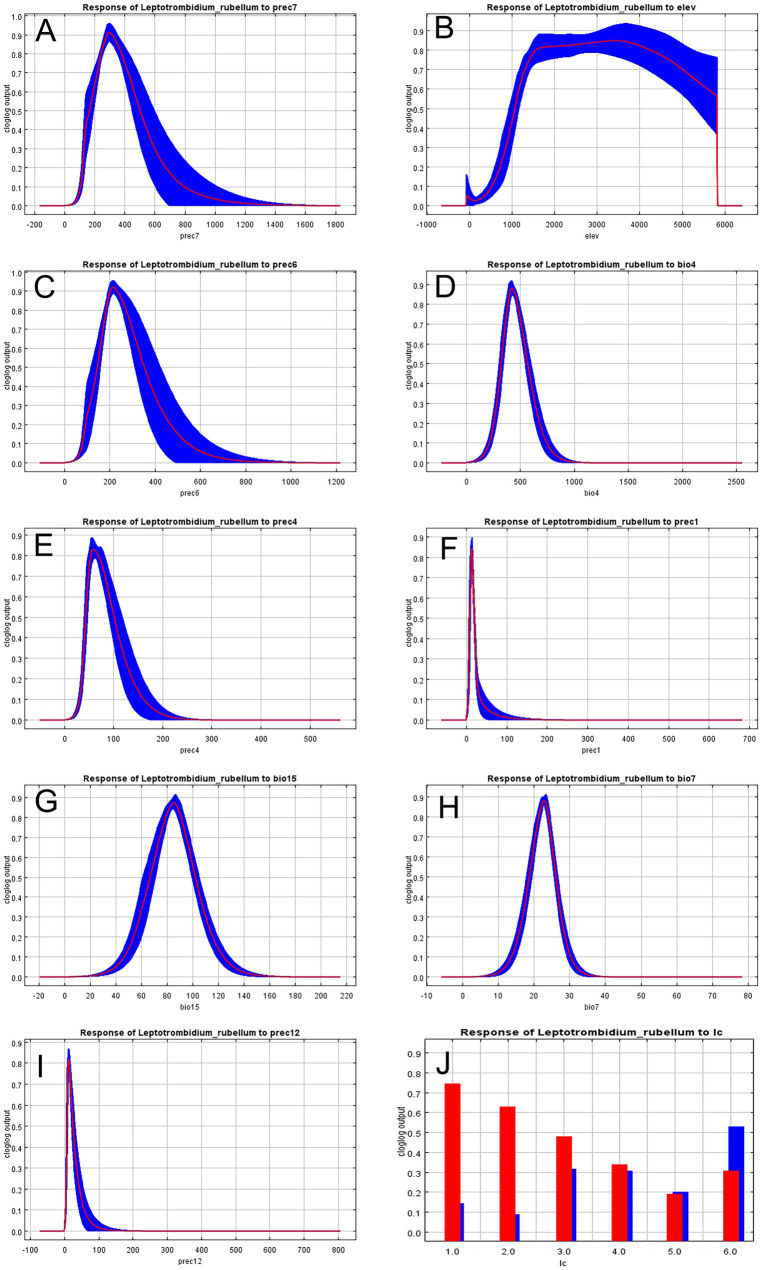
Response curves of climatic variables to the distribution probability of *L. rubellum*. **(A)** Precipitation of July (prec7); **(B)** Elevation (elev); **(C)** Precipitation of June (prec6); **(D)** Temperature seasonality (bio4); **(E)** Precipitation of April (prec4); **(F)** Precipitation of January (prec1); **(G)** Precipitation seasonality (bio15); **(H)** Annual temperature range (bio7); **(I)** Precipitation of December (prc12); **(J)** Land cover type (lc).

Jackknife analysis identified precipitation in July (prec7) as the most influential environmental variable affecting *L. rubellum* distribution, containing unique information not present in other factors, followed by June precipitation (prec6) (Figure 5). Response curves derived from 20 model replicates (with blue margins indicating ±1 SD) revealed specific environmental thresholds: optimal precipitation (prec7) ranged 43.32–1162.87 mm, elevation tolerance spanned −92.50 to 5798.14 m, and land cover suitability followed the hierarchy: croplands > forests > grasslands > urban areas > water bodies > barren lands (Figure 6).

### The potential distribution areas of *Leptotrombidium rubellum* under near current environmental conditions

**Figure 7:**
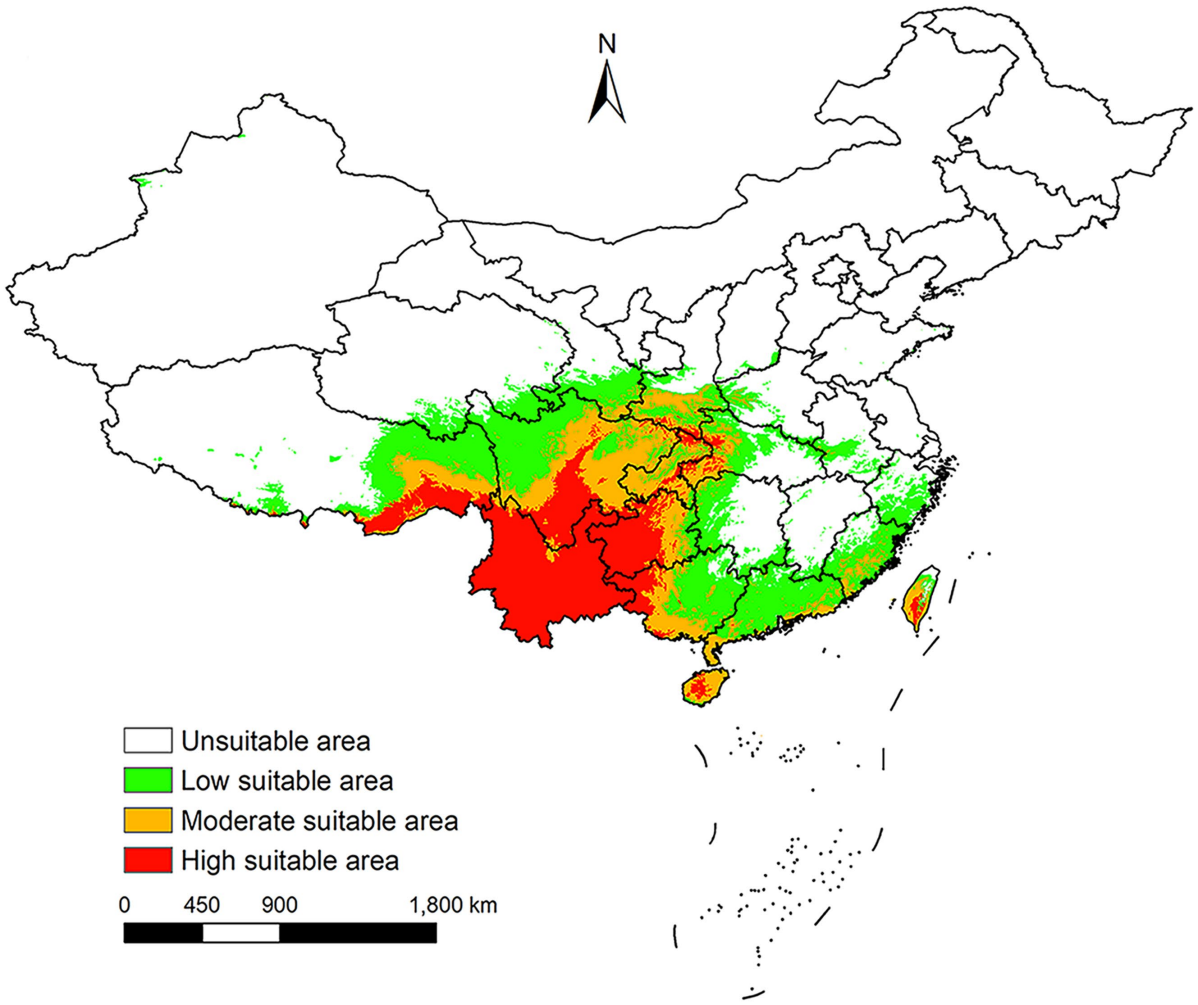
China potential distribution areas of *L. rubellum* under near current climatic conditions.

Under near current conditions, *L. rubellum* is mainly found in southwestern and southeastern China, covering 19 provincial-level regions from Yunnan to Taiwan (Figure 7). Future projections suggest a general expansion of its potential distribution areas, though the extent varies across different scenarios.

### The range of potential distribution areas for *Leptotrombidium rubellum* under future environmental conditions

**Figure 8:**
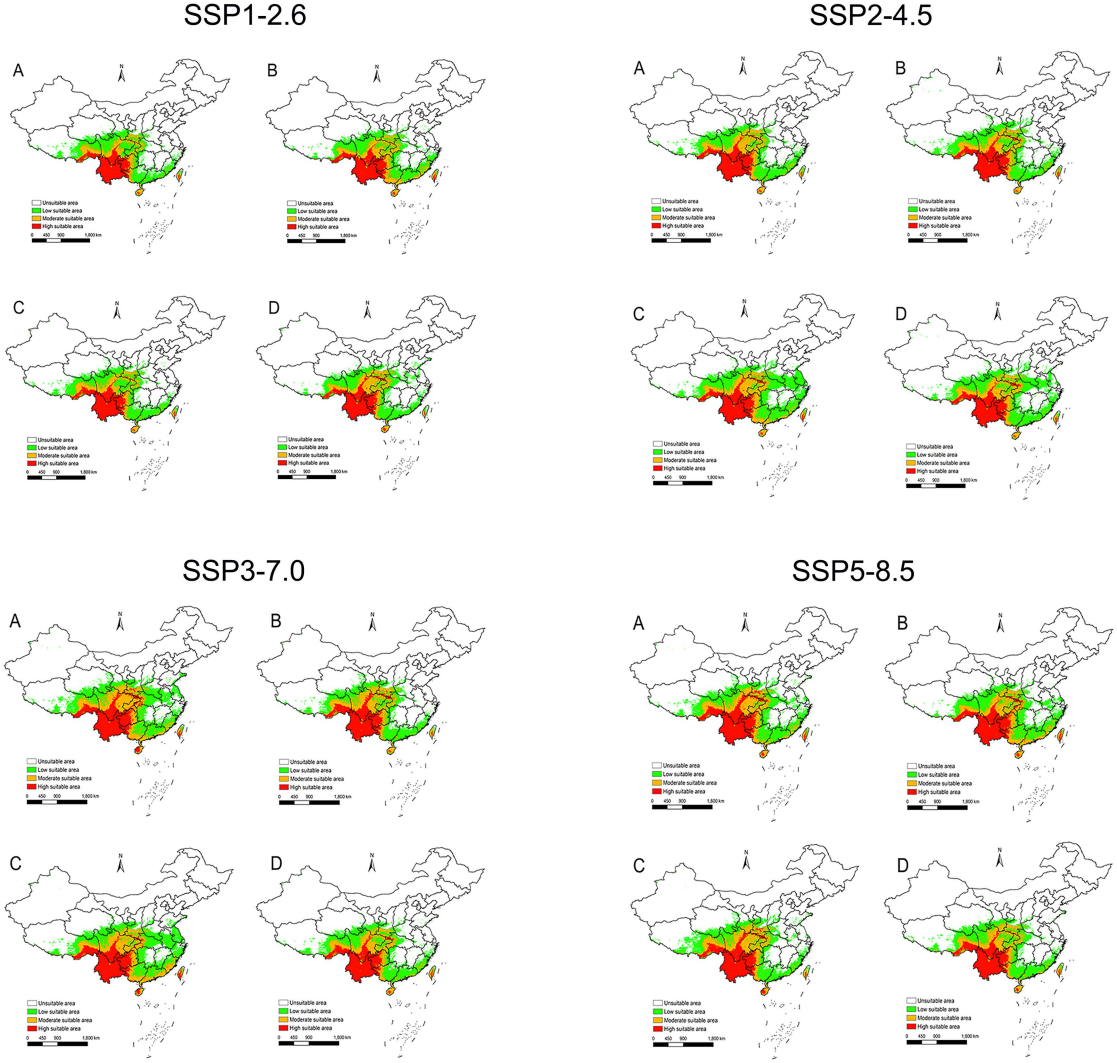
The potential distribution areas of suitable areas for *L. rubellum* around in China under the climatic conditions of SSP1-2.6, SSP2-4.5, SSP3-7.0 and SSP5-8.5. **(A)** 2021–2040; **(B)** 2041–2060; **(C)** 2061–2080; **(D)** 2081–2100.

Under the SSP1-2.6 scenario, the potential distribution areas contracted, decreasing by 3.13% during 2021–2040 and 0.83% during 2061–2080. In contrast, the other scenarios (SSP2-4.5, SSP3-7.0, and SSP5-8.5) consistently projected expansion of suitable areas throughout 2021–2,100 (Figure 8), with increases ranging from 0.00073 to 26.53% (Table 2).

**Table 2:** Current and future potential distribution areas for *L. rubellum* across in China under different climatic conditions.

Climate scenario	Period	Unsuitable area (×10^5^ km^2^)	Moderate suitable area (×10^5^ km^2^)	High suitable area (×10^5^ km^2^)	Total area (×10^5^ km^2^)	Area change (×10^5^ km^2^)	Area change ratio (%)
current	1970–2000	13.24	7.58	8.43	29.25	0.00	
SSP1-2.6	2021–2040	14.29	6.70	7.34	28.34	−0.92	−3.13
	2041–2060	13.81	8.52	7.78	30.11	0.85	2.92
	2061–2080	14.34	6.91	7.76	29.01	−0.24	−0.83
	2081–2100	13.83	7.25	8.17	29.25	0.00	0.00073
SSP2-4.5	2021–2040	13.92	7.82	8.05	29.79	0.54	1.84
	2041–2060	14.97	7.75	8.08	30.80	1.55	5.28
	2061–2080	18.50	9.18	8.37	36.05	6.80	23.23
	2081–2100	17.70	7.84	8.08	33.62	4.37	14.94
SSP3-7.0	2021–2040	16.68	10.41	9.92	37.01	7.76	26.53
	2041–2060	12.93	8.25	8.73	29.91	0.66	2.25
	2061–2080	17.29	10.33	9.28	36.90	7.65	26.14
	2081–2100	14.02	7.97	8.07	30.06	0.80	2.75
SSP5-8.5	2021–2040	15.04	8.97	9.60	33.61	4.36	14.90
	2041–2060	14.41	8.90	8.48	31.78	2.53	8.64
	2061–2080	14.67	8.78	9.07	32.53	3.28	11.20
	2081–2100	13.96	7.93	7.97	29.86	0.61	2.09

**Figure 9:**
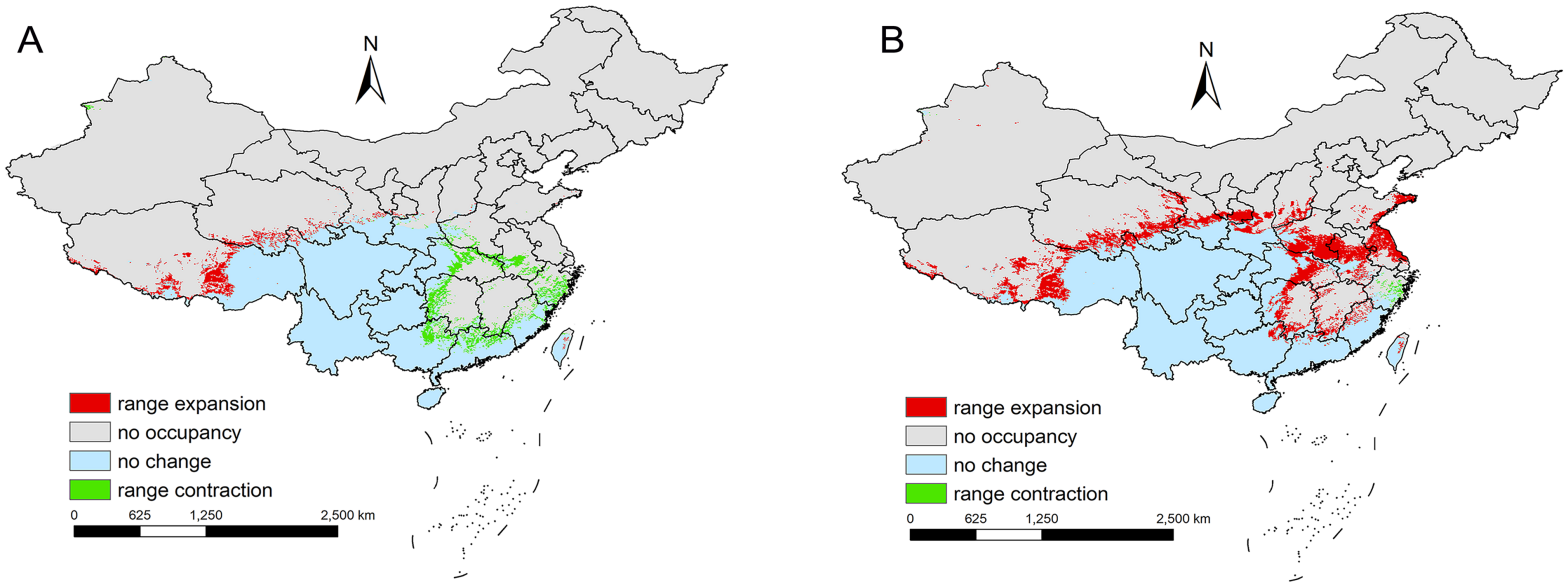
**(A)** Comparison of the current potential distribution areas to SSP1-2.6 2021–2040 periods in China. **(B)** Comparison of the current potential distribution areas to SSP3-7.0 2021–2040 periods in China.

Spatial changes also differ by scenario. For SSP1-2.6 during 2021–2040, there is a reduction of 0.92 × 10^5^ km^2^ primarily in eastern provinces such as Zhejiang to Henan (Figure 9A). In contrast, under SSP3-7.0 during the same period, *L. rubellum* suitable areas expands by 7.76 × 10^5^ km^2^, spreading from Taiwan to Tibet and covering northern and western regions (Figure 9B). The extreme range sizes vary between scenarios, with the smallest being 28.34 × 10^5^ km^2^ under SSP1-2.6 and the largest reaching 37.01 × 10^5^ km^2^ under SSP3-7.0 (Table 2).

## Discussion

With the rising incidence of scrub typhus globally, increasing research attention has been focused on its vector mites. *L. rubellum*, a recognized vector species in China, remains insufficiently studied regarding its geographical distribution. This study employs the MaxEnt model to predict *L. rubellum* potential suitable areas, thereby enhancing our understanding of its distribution patterns and providing valuable insights for public health strategies targeting scrub typhus prevention and control.

The distribution of *L. rubellum* is shaped by multiple environmental variables. As chigger mites progress through seven life stages (egg, deutovum, larva, nymphochrysalis, nymph, imagochrysalis, and adult), with only the larval stage being ectoparasite (31), their extensive free-living phases require adaptation to both abiotic (humidity, soil moisture, temperature) and biotic factors (vegetation density, host interactions). During blood-feeding, *L. rubellum* enters metabolic suppression, becoming particularly sensitive to ambient conditions while relying on vegetation microhabitats for environmental regulation (11). Climate influences not only mite survival but also developmental rates and host-seeking behavior (32).

Climatic variables emerged as primary determinants of *L. rubellum* potential distribution areas, accounting for over 70% of total influence, with July precipitation (prec7) showing particular importance. This likely reflects moisture-dependent physiological requirements during critical midsummer periods. Elevation contributed approximately 26% to distribution patterns, consistent with previous reports of optimal suitability at lower latitudes and elevations (10). Notably, croplands—frequently associated with human activity—represented the most suitable land cover type, suggesting anthropogenic influences on distribution patterns.

Historically confined to coastal Fujian Province (9, 10), *L. rubellum* has recently expanded into Yunnan Province, indicating potential ongoing range shifts. Current suitable areas align with tropical/subtropical rainforests and monsoon climates, concentrated in southwestern and southeastern China including Sichuan, Guizhou, and Taiwan. While a 2022 BRT model produced congruent results (3), that study’s inclusion of multiple mite species may have masked *L. rubellum*-specific patterns and lacked future projections.

Future climate scenarios uniformly predict range expansion for *L. rubellum*, filling a critical knowledge gap as no previous studies have modeled its distribution under climate change (19, 23, 27, 33). Subtropical monsoon regions, highland areas, and zones with active tourism/trade—both coastal and inland—represent priority monitoring areas given invasion risks.

The current analysis has certain limitations that should be acknowledged. First, *L. rubellum* occurrence records were collected post-2001, standardized bioclimatic variables for the 2000–2020 period matching these records are not publicly available through established repositories. Consequently, our models relied on the 1970–2000 climate baseline-the only globally consistent dataset for species distribution modeling under climate change scenarios. Future studies should prioritize integrating dynamically downscaled climate data when accessible. Second, the exclusion of human footprint indices and host interactions, despite their established importance in chigger mite distribution (3, 34), represents a notable constraint. Additionally, the model did not incorporate dynamic ecological processes such as host migration patterns or extreme climate events, nor did it account for short-term climate anomalies that may influence local habitat suitability. Future research addressing these factors would significantly improve the accuracy of distribution forecasting for *L. rubellum*.

## Conclusion

This study provides the first comprehensive prediction of *L. rubellum* potential distribution across China under both current and future environmental conditions. Its primary strengths derive from comprehensive data availability and unique findings that advance understanding of this vector’s distribution patterns. While the MaxEnt model effectively identified key environmental drivers, particularly precipitation and elevation as critical determinants of survival probability, certain limitations should be noted, including potential biases in occurrence data, uncertainties in climate projections, and discrepancies when comparing results with existing findings from eastern regions.

## Data Availability

The original contributions presented in the study are included in the article/Supplementary material, further inquiries can be directed to the corresponding author.

## Footnotes

^1^ https://worldclim.org/

## References

[1] Elliott I Pearson I Dahal P Thomas NV Roberts T Newton PN. Scrub typhus ecology: a systematic review of Orientia in vectors and hosts. Parasit Vectors. (2019) 12:513. doi: 10.1186/s13071-019-3751-x, PMID: 31685019 PMC6829833

[2] Ma T Hao M Chen S Ding F. The current and future risk of spread of *Leptotrombidium deliense* and *Leptotrombidium scutellare* in mainland China. Sci Total Environ. (2022) 843:156986. doi: 10.1016/j.scitotenv.2022.156986, PMID: 35772555

[3] Wang T Meng F Che T Chen J Zhang H Ji Y. Mapping the distributions of blood-sucking mites and mite-borne agents in China: a modeling study. Infect Dis Poverty. (2022) 11:41. doi: 10.1186/s40249-022-00966-0, PMID: 35397554 PMC8994071

[4] Han L Sun Z Li Z Zhang Y Tong S Qin T. Impacts of meteorological factors on the risk of scrub typhus in China, from 2006 to 2020: a multicenter retrospective study. Front Microbiol. (2023) 14:1118001. doi: 10.3389/fmicb.2023.1118001, PMID: 36910234 PMC9996048

[5] Yue Y Ren D Liu X Wang Y Liu Q Li G. Spatio-temporal patterns of scrub typhus in mainland China, 2006-2017. PLoS Negl Trop Dis. (2019) 13:e0007916. doi: 10.1371/journal.pntd.0007916, PMID: 31790406 PMC6917297

[6] Zaman K. Scrub typhus, a salient threat: needs attention. PLoS Negl Trop Dis. (2023) 17:e0011427. doi: 10.1371/journal.pntd.0011427, PMID: 37384603 PMC10309619

[7] Song WY Lv Y Yin PW Yang YY Guo XG. Suitable areas of *Leptotrombidium scutellare* in Yunnan and Sichuan provinces, China, and its association with mite-borne disease transmission. Parasit Vectors. (2023) 16:164. doi: 10.1186/s13071-023-05789-y, PMID: 37194039 PMC10190071

[8] Huang XD Cheng P Zhao YQ Li WJ Zhao JX Liu HM. Chigger mite (Acari: Trombiculidae) survey of rodents in Shandong Province, northern China. Korean J Parasitol. (2017) 55:555–9. doi: 10.3347/kjp.2017.55.5.555, PMID: 29103271 PMC5678473

[9] Geng ML Guo XG Guo B. Geographical distribution and host selection of *Leptotrombidium rubellum* in some parts of Yunnan province. Zhonghua Liu Xing Bing Xue Za Zhi. (2013) 34:152–6. doi: 10.3760/cma.j.issn.0254-6450.2013.02.011, PMID: 23751471

[10] Peng PY Guo XG Jin DC Dong WG Qian TJ Qin F. New record of the scrub typhus vector, *Leptotrombidium rubellum*, in Southwest China. J Med Entomol. (2017) 54:1767–70. doi: 10.1093/jme/tjx133, PMID: 28981880

[11] Ding F Wang Q Hao M Maude RJ John Day NP Lai S. Climate drives the spatiotemporal dynamics of scrub typhus in China. Glob Chang Biol. (2022) 28:6618–28. doi: 10.1111/gcb.16395, PMID: 36056457 PMC9825873

[12] Li X Wei X Yin W Soares Magalhaes RJ Xu Y Wen L. Using ecological niche modeling to predict the suitable areas of scrub typhus in Fujian Province, China. Parasit Vectors. (2023) 16:44. doi: 10.1186/s13071-023-05668-6, PMID: 36721181 PMC9887782

[13] Johnston FH Williamson G Borchers-Arriagada N Henderson SB Bowman DMJS. Climate change, landscape fires, and human health: a global perspective. Annu Rev Public Health. (2024) 45:295–314. doi: 10.1146/annurev-publhealth-060222-034131, PMID: 38166500

[14] Parums DV. A review of the increasing global impact of climate change on human health and approaches to medical preparedness. Med Sci Monit. (2024) 30:e945763. doi: 10.12659/MSM.945763, PMID: 38988000 PMC11302257

[15] Wu Z Wang W Zhu W Zhang P Chang R Wang G. Shrub ecosystem structure in response to anthropogenic climate change: a global synthesis. Sci Total Environ. (2024) 953:176202. doi: 10.1016/j.scitotenv.2024.176202, PMID: 39265690

[16] Borde JP Kaier K Hehn P Matzarakis A Frey S Bestehorn M. The complex interplay of climate, TBEV vector dynamics and TBEV infection rates in ticks-monitoring a natural TBEV focus in Germany, 2009-2018. PLoS One. (2021) 16:e0244668. doi: 10.1371/journal.pone.0244668, PMID: 33411799 PMC7790265

[17] Hekimoglu O Elverici C Kuyucu AC. Predicting climate-driven distribution shifts in *Hyalomma marginatum* (Ixodidae). Parasitology. (2023) 150:883–93. doi: 10.1017/S0031182023000689, PMID: 37519234 PMC10577666

[18] Marina R Ariati J Anwar A Astuti EP Dhewantara PW. Climate and vector-borne diseases in Indonesia: a systematic literature review and critical appraisal of evidence. Int J Biometeorol. (2023) 67:1–28. doi: 10.1007/s00484-022-02390-3, PMID: 36367556

[19] Li C Gao Y Chang N Ma D Zhou R Zhao Z. Risk assessment of *Anopheles philippinensis* and *Anopheles nivipes* (Diptera: Culicidae) invading China under climate change. Biology (Basel). (2021) 10:998. doi: 10.3390/biology10100998, PMID: 34681097 PMC8533129

[20] Li H Liang Y Dong L Li C Zhang L Wang B. Predicting global suitable areas of *Peromyscopsylla hesperomys* and *Orchopeas sexdentatus* and risk assessment for invading China under climate change. Front Public Health. (2023) 10:1018327. doi: 10.3389/fpubh.2022.1018327, PMID: 36684875 PMC9850084

[21] Zhou R Gao Y Chang N Gao T Ma D Li C. Projecting the suitable areas of *Glossinamorsitans* (Diptera: Glossinidae) under climate change using the MaxEnt model. Biology (Basel). (2021) 10:1150. doi: 10.3390/biology10111150, PMID: 34827144 PMC8615152

[22] Cao YT Lu ZP Gao XY Liu ML Sa W Liang J. Maximum entropy Modeling the distribution area of *Morchella* dill. Ex Pers. species in China under changing climate. Biology (Basel). (2022) 11:1027. doi: 10.3390/biology11071027, PMID: 36101408 PMC9312065

[23] Ma D Lun X Li C Zhou R Zhao Z Wang J. Predicting the potential global distribution of *Amblyomma americanum* (Acari: Ixodidae) under near current and future climatic conditions, using the maximum entropy model. Biology (Basel). (2021) 10:1057. doi: 10.3390/biology10101057, PMID: 34681156 PMC8533137

[24] Zhang L Ma D Li C Zhou R Wang J Liu Q. Projecting the suitable areas areas of *Ixodes scapularis* (Acari: Ixodidae) driven by climate change. Biology (Basel). (2022) 11:107. doi: 10.3390/biology11010107, PMID: 35053104 PMC8773098

[25] Arenas-Castro S Sillero N. Cross-scale monitoring of habitat suitability changes using satellite time series and ecological niche models. Sci Total Environ. (2021) 784:147172. doi: 10.1016/j.scitotenv.2021.147172 34088022

[26] Dakhil MA El-Keblawy A El-Sheikh MA Halmy MWA Ksiksi T Hassan WA. Global invasion risk assessment of *Prosopis juliflora* at biome level: does soil matter? Biology (Basel). (2021) 10:203. doi: 10.3390/biology10030203, PMID: 33803081 PMC7999975

[27] Ji H Wei X Ma D Wang X Liu Q. Predicting the global suitable areas of two major vectors of Rocky Mountain spotted fever under conditions of global climate change. PLoS Negl Trop Dis. (2024) 18:e0011883. doi: 10.1371/journal.pntd.0011883, PMID: 38198451 PMC10805312

[28] Salvà-Catarineu M Romo A Mazur M Zielińska M Minissale P Dönmez AA. Past, present, and future geographic range of the relict Mediterranean and Macaronesian *Juniperus phoenicea* complex. Ecol Evol. (2021) 11:5075–95. doi: 10.1002/ece3.7395, PMID: 34025993 PMC8131820

[29] Escobar LE Lira-Noriega A Medina-Vogel G Townsend PA. Potential for spread of the white-nose fungus (Pseudogymnoascus destructans) in the Americas: use of MaxEnt and NicheA to assure strict model transference. Geospat Health. (2014) 9:221–9. doi: 10.4081/gh.2014.19, PMID: 25545939

[30] Soucy JR Slatculescu AM Nyiraneza C Ogden NH Leighton PA Kerr JT. High-resolution ecological niche Modeling of *Ixodes scapularis* ticks based on passive surveillance data at the northern frontier of Lyme disease emergence in North America. Vector Borne Zoonotic Dis. (2018) 18:235–42. doi: 10.1089/vbz.2017.2234, PMID: 29565748 PMC5930794

[31] Lv Y Guo XG Jin DC. Research Progress on Leptotrombidium deliense. Korean J Parasitol. (2018) 56:313–24. doi: 10.3347/kjp.2018.56.4.313, PMID: 30196663 PMC6137299

[32] Seto J Suzuki Y Nakao R Otani K Yahagi K Mizuta K. Meteorological factors affecting scrub typhus occurrence: a retrospective study of Yamagata prefecture, Japan, 1984-2014. Epidemiol Infect. (2017) 145:462–70. doi: 10.1017/S0950268816002430, PMID: 27788693 PMC9507639

[33] Li F Mu Q Ma D Wu Q. Predicting the potential global distribution of *Ixodes pacificus* under climate change. PLoS One. (2024) 19:e0309367. doi: 10.1371/journal.pone.0309367, PMID: 39190767 PMC11349213

[34] Skinner EB Glidden CK MacDonald AJ Mordecai EA. Human footprint is associated with shifts in the assemblages of major vector-borne diseases. Nat Sustain. (2023) 6:652–61. doi: 10.1038/s41893-023-01080-1, PMID: 37538395 PMC10399301

